# The Incidence of Venous Thromboembolism in Critically Ill Patients with SARS-CoV-2 Infection Compared with Critically Ill Influenza and Community-Acquired Pneumonia Patients: A Retrospective Chart Review

**DOI:** 10.3390/medsci10020030

**Published:** 2022-06-08

**Authors:** Sean Boyd, Kai Sheng Loh, Jessie Lynch, Dhari Alrashed, Saad Muzzammil, Hannah Marsh, Mustafa Masoud, Salman Bin Ihsan, Ignacio Martin-Loeches

**Affiliations:** 1 Multidisciplinary Intensive Care Research Organization (MICRO), St James’s Hospital, D08 NHY1 Dublin, Ireland; lohk@tcd.ie (K.S.L.); jessielynch993@gmail.com (J.L.); dharialrashed@rcsi.com (D.A.); drsaadsheikh@gmail.com (S.M.); hannahmarsh@rcsi.ie (H.M.); mustesharef@gmail.com (M.M.); binihsas@tcd.ie (S.B.I.); drmartinloeches@gmail.com (I.M.-L.); 2 Medicine, Trinity College Dublin, University of Dublin, DN02 PN40 Dublin, Ireland; 3 Pulmonary Intensive Care Unit, Respiratory Institute, Hospital Clinic of Barcelona, IDIBAPS (Institut d’Investigacions Biomèdiques August Pi i Sunyer), University of Barcelona, CIBERes, 08036 Barcelona, Spain

**Keywords:** VTE (venous thromboembolism), PE (pulmonary embolism), DVT (deep vein thrombosis), ICU (intensive care unit), COVID-19, influenza, community-acquired pneumonia

## Abstract

The rate of venous thromboembolism in COVID-19 patients has been reported to be 30% (deep vein thrombosis 20% and pulmonary embolism 18%). This has been shown to be higher in COVID-19 patients admitted to the ICU. Prophylactic anticoagulation may be sufficient at ward level, but not in intensive care. A retrospective chart review was undertaken in a large university hospital. The review included 276 patients from COVID-19 Wave 1, COVID-19 Wave 2, influenza, and community-acquired pneumonia groups. The timeframe included patients admitted between 23 February 2014 and 12 May 2021. Clinical characteristics, outcomes, blood results, rates of venous thromboembolism, and anticoagulation status were recorded. The incidence of venous thromboembolism in COVID-19 Wave 1, COVID-19 Wave 2, influenza, and community-acquired pneumonia was 10.91%, 13.69%, 13.33%, and 6.81%, respectively (*p* = 0.481). The incidence of pulmonary embolism was 7.27%, 10.95%, 3.33%, and 5.68%, respectively (*p* = 0.350). The incidence of deep vein thrombosis was 5.45%, 5.48%, 10.00%, and 1.14%, respectively (*p* = 0.117). Although most patients were prophylactically anticoagulated, venous thromboembolism still occurred. Venous thromboembolism remains an important differential to consider in critically ill COVID-19 patients. The current literature does not advise therapeutic anticoagulation for thromboprophylaxis in the ICU.

## 1. Introduction

The COVID-19 pandemic has been ongoing for over two years and has brought along a host of new challenges posed to clinicians. Venous thromboembolism is no different. A meta-analysis in October 2020 reported an approximate venous thromboembolism rate of 30% in this cohort (deep vein thrombosis 20% and pulmonary embolism 18%) [1]. When comparing COVID-19 patients treated on the ward and in intensive care, elevated rates have been reported, of 25% and 31%, respectively [2,3,4]. In contrast, more recent studies have reported venous thromboembolism rates in the ICU (intensive care unit) as low as 4.9% [5]. It is apparent that a wide variation in the literature exists.

When comparing COVID-19 patients to non-COVID-19 patients, an elevated risk of venous thromboembolism has been shown. It has been reported that critically ill COVID-19 patients have a 16% higher risk of developing venous thromboembolism when compared to non-COVID-19 patients [6]. There are several possible explanations for this. COVID-19 may be related to coagulopathy [7,8,9,10,11]. Elevated D-dimer and fibrinogen are among the most common findings [12]. SARS-CoV-2 infection has also been reported to cause pulmonary vascular endothelial inflammation and subsequent thrombosis [13,14,15,16,17,18]. It has been described as causing an inflammatory, prothrombotic, hypofibrinolytic state [19,20]. However, this is possibly the case in all viral infections [21]. A smaller study was carried out at our institution last year, which compared critically ill COVID-19 patients, influenza patients, and a control group of patients intubated for non-infective reasons. It was shown that there was no significant difference in the rate of venous thromboembolism between the COVID-19 group and the influenza group. Both groups, however, showed a higher rate than the control group [22].

A recent meta-analysis by Lobbes et al. looked at risk factors for venous thromboembolism development in severe COVID-19 infections [23]. They found moderate-certainty evidence for an association with D-dimer peaks, duration of hospitalisation, and duration of intubation. They found low-certainty evidence for C-reactive protein, D-dimer (not peak), troponin, and the requirement for inotropic support [23].

Anticoagulation strategies pose a challenge to clinicians when treating this cohort. It has been suggested that although prophylactic anticoagulation may be sufficient on the ward, it may not provide adequate cover for ICU patients [5,24]. A randomised control trial showed a significant difference in the rate of major venous thromboembolism in hospitalised COVID-19 patients treated with therapeutic doses of anticoagulants versus prophylactic doses [25]. No significant increase in major haemorrhagic events was shown. *This did not include critically ill COVID-19 patients*. The ongoing ACTIV (Accelerating COVID-19 Therapeutic Interventions and Vaccines) trial has shown that therapeutic dosing of anticoagulants is superior to prophylactic dosing in preventing ICU admission for ventilation or organ support in *hospitalised patients on the ward*. In comparison, this study has shown that therapeutic dosing in *ICU patients* is not beneficial and is potentially harmful [26,27]. In the absence of contraindications, therapeutic anticoagulation is advised in the treatment of venous thromboembolism, similar to non-COVID-19 patients [28]. A narrative review of the latest evidence by Angelini et al. concludes that further research is required regarding risk stratification, optimal anticoagulation dosing, and extended prophylaxis for venous thromboembolism in COVID-19 patients [28].

Overall, venous thromboembolism remains a pertinent differential to keep in mind when treating this group.

## 2. Materials and Methods

A retrospective chart review was performed including 276 patients who were admitted to ICU in a large university hospital in Ireland. The following four groups were collected:

1. The first wave of COVID-19, admitted between 15 March 2020 and 24 May 2020 (n = 55).
2. The second wave of COVID-19 admitted between 31 August 2020 and 13 February 2021 (n = 73).
3. All influenza patients admitted between 23 February 2014 and 3 February 2020 (n = 60).
4. All community-acquired pneumonia patients admitted between 11 January 2019 and 12 May 2021 (n = 88).

Only patients that were admitted to the ICU were included. All patients that were included in both COVID-19 groups had a positive polymerase chain reaction for SARS-CoV-2. Exact strains were not determined. This is a limitation of this study. All patients included in the influenza group had a positive polymerase chain reaction for influenza A or B. The community-acquired pneumonia cohort was included based on clinical diagnosis. The influenza and community-acquired pneumonia groups were collected as far back as the instatement of the hospital’s electronic record system. This was to ensure similar numbers in each group.

The aim of this study was to compare the rates of venous thromboembolism in all four groups.

Each patient’s clinical characteristics were recorded. Severity of illness was expressed through SAPSII (simplified acute physiology) and SOFA (sequential organ failure assessment) scores. Immunosuppression was defined by any one of the following criteria: solid tumour with chemotherapy in the last 3 months, progressive metastatic disease, haematological malignancies, solid organ transplantation, HIV infection (with or without AIDS), corticosteroids (>3 months at any dosage or ≥1 mg/kg prednisone equivalent per day for >7 days), and immunosuppressive drugs.

The rate of venous thromboembolism, including deep vein thrombosis and pulmonary embolism, was recorded for each group. Venous thromboembolism was only recorded if it was confirmed on imaging. This included computed tomography pulmonary angiogram, and ultrasound. On some occasions, venous thromboembolism was identified incidentally. For example, pulmonary embolism may have been identified by computed tomography of the abdomen and pelvis.

Each patient’s coagulation profile and blood results from admission to ICU were recorded. This point in time was chosen as it provides a fair comparison between all groups. It removes confounders such as ICU length of stay, variable length of ICU anticoagulation and length of mechanical ventilation. D-dimers were unavailable, as not indicated in many situations. If venous thromboembolism was suspected in this critically ill cohort, D-dimer provided minimal aid in diagnosis.

We used SPSS (version 20) for data analysis. All *p*-values were two-tailed. We considered differences as significant if *p* was less than 0.05. We reported categorical variables as numbers and frequencies (%), normally distributed continuous variables as mean and standard deviation [SD], and skewed continuous variables as median and interquartile range [IQR]. We performed χ2 tests and Fisher’s exact tests to compare qualitative variables and Student’s *t*-tests and ANOVAs (analysis of variance) or Mann–Whitney U and non-parametric Kruskal–Wallis tests to compare normally distributed or skewed continuous variables, whenever appropriate.

This study has been deemed as minimal-risk research using data collected for routine clinical practice; therefore, the requirement for informed consent has been waived. Reference: REC: 2020-05 List 17.

## 3. Results

### 3.1. Clinical Characteristics and Outcomes

The clinical characteristics and outcomes are displayed in Table 1. There was a male predominance throughout all four groups (*p* = 0.381). The mean age, co-morbidities, and BMI (body mass index) were similar throughout all four groups, apart from the following. The rate of ischaemic heart disease was significantly elevated in the influenza group (*p* < 0.001). The rate of hypertension was significantly elevated in the COVID-19 Wave 2 group (*p* = 0.012). The rate of chronic obstructive pulmonary disease was significantly elevated in the influenza group and the community-acquired pneumonia group compared to both COVID-19 groups (*p* = 0.003).

The mean SAPSII scores [SD] were 49.75 [18.63], 41.63 [17.63], 55.73 [17.26], and 48.92 [18.83], respectively (*p* < 0.001). The mean SOFA scores [SD] were 9.18 [4.32], 8.94 [4.77], 11.13 [4.49], and 9.76 [4.15], respectively (*p* = 0.029). A significant elevation in both SAPSII and SOFA scores was seen in the influenza group.

The mean ICU length of stay (*p* = 0.010), and length of mechanical ventilation (*p* = 0.009) were significantly increased in the second wave of COVID-19 compared to the first wave. ICU mortality was significantly increased in both the COVID-19 Wave 2 group and the influenza group compared to the other groups (*p* = 0.047).

### 3.2. Blood Results on Admission

Blood results from admission are displayed in Table 2. All groups displayed an elevated mean PT (prothrombin time), fibrinogen, C-reactive protein and LDH (lactate dehydrogenase). aPTT (activated partial thromboplastin time) and platelets were within the normal range throughout all groups. The influenza group displayed a significantly lower fibrinogen level (*p* = 0.003) and platelet count than the other groups (*p* = 0.026). The COVID-19 Wave 2 group had a significantly lower C-reactive protein level than the other groups (*p* = 0.012). A significantly elevated LDH was also found in the influenza group (*p* < 0.001).

### 3.3. Rates of Venous Thromboembolism, Deep Vein Thrombosis, and Pulmonary Embolism

The rates of venous thromboembolism, deep vein thrombosis, and pulmonary embolism are shown in Table 3. No significant difference was found between the four groups.

### 3.4. Prophylactic and Therapeutic Anticoagulation

The majority of patients received prophylactic anticoagulation in the form of low molecular weight heparin (subcutaneous enoxaparin) from admission to ICU. Some patients were therapeutically anticoagulated with a heparin infusion for other reasons, such as atrial fibrillation or due to suspicion of undiagnosed venous thromboembolism. A few were not anticoagulated due to increased bleeding risk.

In COVID-19 Wave 1 (n = 55), 49 patients (89.09%) were prophylactically anticoagulated and 5 (9.09%) were therapeutically anticoagulated from admission. One patient (1.82%) did not receive anticoagulation due to increased bleeding risk and did not develop venous thromboembolism. In COVID-19 Wave 2 (n = 73), 55 patients (75.34%) were prophylactically anticoagulated and 13 (17.81%) were therapeutically anticoagulated from admission. Five patients (6.85%) were not anticoagulated due to a high bleeding risk. No episodes of venous thromboembolism were recorded in the patients that did not receive anticoagulation. In the influenza group (n = 60), 41 patients (68.33%) were prophylactically anticoagulated and 12 (20.00%) were therapeutically anticoagulated from admission. Seven patients (11.67%) were not anticoagulated due to a high bleeding risk. Of these, one patient developed a deep vein thrombosis. In the community-acquired pneumonia group (n = 88), 59 patients (67.05%) were prophylactically anticoagulated and 19 (21.59%) were therapeutically anticoagulated from admission. Ten patients (11.36%) were not anticoagulated due to a high bleeding risk, including one patient who declined anticoagulation. No episodes of venous thromboembolism were recorded in the patients that did not receive anticoagulation.

## 4. Discussion

In our ICU, no significant difference was found regarding the rate of venous thromboembolism between all four groups. Several studies have examined the rate of venous thromboembolism in the ICU before the COVID-19 pandemic. Deep vein thrombosis rates have been described between 10% and 37% [29,30,31,32,33]. Pulmonary embolism rates have been shown to be much lower. Variable rates of venous thromboembolism in COVID-19 patients have been reported (4.9% to 30%) [1,2,3,4,34,35]. It has been described that the risk of venous thromboembolism is increased by 16% in COVID-19 patients compared to non-COVID-19 patients in ICU [6].

A smaller study performed at our institution showed there was no significant difference in the rate of venous thromboembolism between critically ill COVID-19 and influenza patients. Yet, the rate was higher in both groups than in the non-viral control group [22]. In the current study, similar venous thromboembolism rates were reported in critically ill COVID-19 patients. Higher rates of venous thromboembolism were displayed in the COVID-19 and influenza groups when compared to the community-acquired pneumonia group, but not significantly so (*p* = 0.481). Notably, pulmonary embolism was more frequently reported in the COVID-19 groups and the community-acquired pneumonia groups (*p* = 0.350). It must be kept in mind that the differences when compared with other studies may be due to population differences, different ICU admission criteria, different thromboprophylaxis regimens, and smaller study numbers.

Moderate-certainty evidence has been reported for an association between development of venous thromboembolism and duration of hospitalisation and duration of intubation [23]. Although a significant increase in ICU length of stay (*p* = 0.010) and length of mechanical ventilation (*p* = 0.009) was found in the COVID-19 Wave 2 group compared to the others, no significant increase in venous thromboembolism was found.

Venous thromboembolism still occurred despite prophylactic anticoagulation, and this has been previously reported [4,5]. Tang et al. reported that COVID-19 patients that receive prophylactic thromboprophylaxis potentially have better outcomes [36,37]. Currently, pharmacological thromboprophylaxis is advised for all COVID-19 patients, unless contraindicated. Mechanical thromboprophylaxis is advised at a minimum [26,27]. Enoxaparin has the potential to bind certain viruses as they pass through the extracellular matrix of the respiratory tract [38]. This may provide additional benefit. The evidence for therapeutic versus prophylactic anticoagulation in COVID-19 patients both on the ward and in the ICU is still evolving and further research is required [28]. In the absence of contraindications, therapeutic anticoagulation is advised in the treatment of venous thromboembolism, similar to non-COVID-19 patients [28]. Nebulised fibrinolytics/anticoagulants may provide improved outcomes in the future, targeting pulmonary microthrombosis or pulmonary embolism. Further research in this area is also required [28,39,40].

Prouse et al. demonstrated that a SOFA score of three or greater was associated with an increased risk of developing deep vein thrombosis [41]. The mean SOFA scores recorded for all four groups in our study were greater than 3. Therefore, we cannot make any comment on association of SOFA score and deep vein thrombosis risk from this study.

Studies have described a prolonged PT in COVID-19 patients [42,43]. This has been linked to increased mortality [7,44]. In this study, a prolonged PT was displayed in all groups. A higher mortality rate was seen in the second wave of COVID-19 and the influenza group. We cannot conclude that prolonged PT was related to increased mortality from this study.

In our study, the mean aPTT in all groups was within the normal range. This contrasts with our previous study, in which the aPTT in all groups was low to normal range [22]. Several publications have investigated the aPTT in COVID-19 patients. Prolonged aPTT may be secondary to antiphospholipid antibodies. Zuo et al. reported eight different antiphospholipid antibodies found in COVID-19 patients that might be responsible for prolonged aPTT [45]. Harzallah et al. reported that 45% of COVID-19 patients were found to be positive for lupus anticoagulant [46]. Lupus anticoagulant has been shown not to be associated with higher rates of venous thromboembolism [47]. Antiphospholipid antibodies may be detected in acute infection [48]. Therefore, the significance of these antibodies is still in question.

Fibrinogen levels and C-reactive protein were elevated throughout all groups. This has been reported in previous studies [49]. The influenza group displayed a significantly lower fibrinogen level when compared to the other groups (*p* = 0.003). One study showed how inflammatory markers may be more associated with venous thromboembolism than the coagulation profile [50]. LDH was shown to be elevated in all groups and was significantly elevated in the influenza group (*p* < 0.001). Yet, no significant difference in venous thromboembolism rate was found. Elevated LDH has been reported to be linked to a poorer prognosis [51]. Although a significant increase in the ICU mortality rate was noted in the second wave of COVID-19 compared to the first (*p* = 0.047), the only significant difference in blood work was a significantly lower C-reactive protein level (*p* = 0.012).

A recent meta-analysis performed by Bhakta et al. [52] examined the rates of venous thromboembolism in Black/African American and White patients. It was concluded that there was no significant difference. Unfortunately, our study did not record the ethnicity of patients, but we would agree that this would be an interesting factor to record and would advise it to be included in future studies.

Venous thromboembolism was only recorded if confirmed by imaging. Imaging was performed only if clinically indicated. Therefore, undiagnosed venous thromboembolism would be excluded. One study has shown the rates of asymptomatic deep vein thrombosis and pulmonary embolism in hospitalised COVID-19 patients to be 25.5% and 24.3%, respectively [53]. This is a limitation of this study.

## 5. Conclusions

In conclusion, the COVID-19 pandemic has created a host of challenges to intensivists across the globe. Venous thromboembolism is no different. At our institution, the rates of venous thromboembolism in ICU in the first wave of COVID-19, the second wave of COVID-19, influenza, and community-acquired pneumonia were 10.91%, 13.69%, 13.33%, and 6.81%, respectively (*p* = 0.481). Similar to a previous study performed at our institution, venous thromboembolism occurred more frequently in critically ill patients with COVID-19 or influenza than in the non-viral group [22]. This difference was not significant. Evidence for anticoagulation strategies is still evolving, and further research is required [28]. The current literature does not advise therapeutic anticoagulation for thromboprophylaxis in ICU at this point in time [26,27]. Despite thromboprophylaxis, venous thromboembolism still occurred in our ICU. Venous thromboembolism remains an important differential to consider in the treatment of critically ill COVID-19 patients.

## Figures and Tables

**Table 1:** Clinical characteristics and outcomes.

Parameters	COVID-19 Wave 1 (n = 55)	COVID-19 Wave 2 (n = 73)	Influenza (n = 60)	Community-Acquired Pneumonia (n = 88)	*p*-Value
n = 276					
Male (n, %)	38 (69.09%)	48 (65.75%)	33 (55.00%)	56 (63.64%)	0.381
Female (n, %)	17 (30.91%)	25 (34.25%)	27 (45.00%)	32 (36.36%)	0.381
Age (years) (mean, [SD])	60.38 [13.65]	64.33 [12.21]	61.70 [16.58]	62.33 [15.13]	0.476
CCF (n, %)	6 (10.90%)	5 (6.85%)	6 (10.00%)	10 (11.36%)	0.791
IHD (n, %)	13 (23.63%)	9 (12.32%)	25 (41.67%)	10 (11.36%)	<0.001
HTN (n, %)	17 (30.90%)	37 (50.68%)	19 (31.67%)	24 (27.27%)	0.012
DM (n, %)	12 (21.82%)	17 (23.29%)	11 (18.33%)	8 (9.09%)	0.079
COPD (n, %)	6 (10.90%)	15 (20.55%)	22 (36.67%)	30 (34.09%)	0.003
Asthma (n, %)	7 (12.73%)	9 (12.32%)	7 (11.67%)	7 (7.95%)	0.757
CKD (n, %)	7 (12.73%)	3 (4.11%)	3 (5.00%)	4 (4.55%)	0.160
Cirrhosis (n, %)	0 (0%)	2 (2.74%)	1 (1.67%)	2 (2.27%)	0.687
Cancer (n, %)	1 (1.82%)	10 (13.70%)	7 (11.67%)	13 (14.77%)	0.092
Immunosuppressed * (n, %)	5 (9.09%)	12 (16.44%)	9 (15.00%)	15 (17.05%)	0.558
BMI (kg/m^2^) (mean, [SD])	29.80 [16.28]	29.18 [6.58]	27.08 [9.62]	25.79 [9.55]	0.412
SAPSII (mean, [SD])	49.75 [18.63]	41.63 [17.63]	55.73 [17.26]	48.92 [18.83]	<0.001
SOFA worst throughout admission (mean, [SD])	9.18 [4.32]	8.94 [4.77]	11.13 [4.49]	9.76 [4.15]	0.029
ICU LOS (median, [IQR])	12.00 [5.00, 26.00]	14.00 [6.00, 32.50]	9.00 [3.25, 20.00]	10.00 [4.00, 18.75]	0.010
MV (median, [IQR])	8.00 [0.00, 17.00]	11.00 [0.50, 25.00]	7.00 [1.00, 14.75]	5.00 [0.25, 14.00]	0.009
ICU mortality (n, %)	9 (16.36%)	28 (38.36%)	20 (33.33%)	23 (26.14%)	0.047

CCF, congestive cardiac failure; IHD, ischaemic heart disease; HTN, hypertension; DM, diabetes mellitus; COPD, chronic obstructive pulmonary disease; CKD, chronic kidney disease, BMI, body mass index; SAPS, Simplified Acute Physiology Score; SOFA, Sequential Organ Failure Assessment; LOS, length of stay; MV, mechanical ventilation. * Immunosuppression criteria used: solid tumour with chemotherapy in the last 3 months, progressive metastatic disease, haematological malignancies, solid organ transplantation, HIV infection (with or without AIDS), corticosteroids (>3 months at any dosage or ≥1 mg/kg prednisone equivalent per day for >7 days), and immunosuppressive drugs.

**Table 2:** Blood results on admission.

Parameters	Normal Range	COVID-19 Wave 1 (n = 55)	COVID-19 Wave 2 (n = 73)	Influenza (n = 60)	Community-Acquired Pneumonia (n = 88)	*p*-Value
n = 276						
PT (seconds) (mean, [SD])	9.9–13.1	14.27 [3.28]	15.22 [6.53]	15.23 [10.47]	15.52 [5.77]	0.763
aPTT (seconds) (mean, [SD])	24.0–36.0	34.58 [17.40]	32.39 [13.34]	33.99 [11.94]	32.45 [7.39]	0.673
Fibrinogen (g/L) (mean, [SD])	1.9–3.5	5.88 [1.60]	5.12 [2.00]	3.67 [1.98]	5.06 [2.39]	0.003
Platelets (×10^9^/L) (mean, [SD])	140–450	260.15 [150.50]	234.81 [104.10]	192.53 [104.64]	244.38 [139.00]	0.026
CRP (mg/L) (mean, [SD])	<10	152.72 [100.1]	101.61 [88.20]	128.19 [104.07]	152.77 [125.51]	0.012
LDH (IU/L) (mean, [SD])	135–250	405.33 [209.14]	516.45 [237.85]	1465.78 [2841.29]	425.56 [257.89]	<0.001

PT, prothrombin time; aPTT, activated partial thromboplastin time; CRP, C-reactive protein; LDH, lactate dehydrogenase.

**Table 3:** Rates of venous thromboembolism, deep vein thrombosis, and pulmonary embolism *.

Parameters	COVID-19 Wave 1 (n = 55)	COVID-19 Wave 2 (n = 73)	Influenza (n = 60)	Community-Acquired Pneumonia (n = 88)	*p*-Value
n = 276					
VTE (n, %)	6 (10.91%)	10 (13.69%)	8 (13.33%)	6 (6.81%)	0.481
PE (n, %)	4 (7.27%)	8 (10.95%)	2 (3.33%)	5 (5.68%)	0.350
DVT (n, %)	3 (5.45%)	4 (5.48%)	6 (10.00%)	1 (1.14%)	0.117

VTE, venous thromboembolism; PE, pulmonary embolism; DVT, deep vein thrombosis. * Some patients had both DVT and PE.

## Data Availability

For queries regarding data availability, please contact the corresponding author.
